# Baseline and dynamic CALLY index changes predict mortality in sepsis-associated acute kidney injury: a two-center retrospective cohort study

**DOI:** 10.3389/fnut.2026.1858222

**Published:** 2026-07-28

**Authors:** Wanyao Lin, Qianping Zhang, Xinyi Tian, Yan Zhang, Kaifan Lin, Bihuan Cheng, Yuqiang Gong, Shengwei Jin, Ye Gao

**Affiliations:** 1 Department of Anesthesia, Pain and Critical Care, The Second Affiliated Hospital of Wenzhou Medical University, Wenzhou, Zhejiang, China; 2 Department of Emergency Medicine, Taizhou Hospital of Zhejiang Province, Taizhou, Zhejiang, China; 3 Ningbo Municipal Hospital of Traditional Chinese Medicine (TCM), Affiliated Hospital of Zhejiang Chinese Medical University, Ningbo, Zhejiang, China; 4 Key Laboratory of Pediatric Anesthesiology, Ministry of Education, Wenzhou Medical University, Wenzhou, Zhejiang, China; 5 Laboratory of Anesthesiology of Zhejiang Province, The Second Affiliated Hospital and Yuying Children's Hospital of Wenzhou Medical University, Wenzhou, Zhejiang, China; 6 NHC Key Lab of Reproduction Regulation, Shanghai Engineering Research Center of Reproductive Health Drug and Devices, Shanghai Institute for Biomedical and Pharmaceutical Technologies, Shanghai, China; 7 Wenzhou Key Laboratory of Precision General Practice and Health Management, Wenzhou, Zhejiang, China

**Keywords:** C-reactive protein-albumin-lymphocyte index, mortality, nutritional-immunological marker, prognostic marker, sepsis-associated acute kidney injury

## Abstract

**Purpose:**

Sepsis-associated acute kidney injury (SA-AKI) is associated with high mortality rates, yet prognostic biomarkers that jointly reflect nutritional, inflammatory and immune status remain limited. This study aimed to assess the prognostic value of the C-reactive protein-albumin-lymphocyte (CALLY) index in this patient population.

**Patients and methods:**

In this retrospective two-center cohort study, 1,019 adult patients with SA-AKI were included. Kaplan–Meier and log-rank analyses identified the Day 3 CALLY index as the primary prognostic variable. Restricted cubic spline (RCS) analysis was used to characterize nonlinear associations with mortality, multivariable Cox proportional hazards models were applied to adjust for potential confounders, exploratory causal mediation analysis were performed to assess the contribution of the CALLY index to age-related mortality risk, and Bayesian joint models were used to evaluate the prognostic value of longitudinal CALLY trajectories.

**Results:**

A higher Day 3 CALLY index was independently correlated with lower 30-day mortality (HR: 0.997, 95% CI: 0.995–0.999) and showed similar associations with 60-day, 365-day, and in-hospital outcomes (all *p* < 0.05). RCS analysis revealed a significant L-shaped nonlinear association with the Day 3 CALLY index and 30-day mortality, with an inflection point of 11.52. Mediation analysis indicated that the CALLY index explained 23.8–28.7% of the association between age and mortality. In Bayesian joint models, the inverse association between the CALLY index and mortality became progressively stronger over time.

**Conclusion:**

The CALLY index, an integrated marker of nutritional, inflammatory, and immune status, was independently associated with mortality in patients with SA-AKI. The Day 3 CALLY index provided clinically relevant prognostic stratification, and its longitudinal changes further improved risk assessment.

## Introduction

Sepsis-associated acute kidney injury (SA-AKI), defined as acute kidney injury developing within seven days of sepsis diagnosis according to KDIGO criteria (1, 2), is a common and severe complication in critically ill patients. It accounts for nearly half of AKI cases in the intensive care unit and is associated with prolonged hospitalization, increased use of renal replacement therapy, and high mortality (3, 4). SA-AKI arises from a complex interplay of systemic inflammation, immune dysregulation, and metabolic stress (5), highlighting the need for biomarkers that reflect these interconnected pathophysiological processes (6).

Current risk stratification tools for SA-AKI remain suboptimal. Traditional functional markers such as serum creatinine are limited by delayed elevation, whereas newer injury biomarkers such as neutrophil gelatinase-associated lipocalin (NGAL) are not yet routinely available for rapid clinical assessment (7). Composite indices based on routine laboratory parameters, including the prognostic nutritional index (8) and controlling nutritional status (CONUT) score (9), provide prognostic information on nutritional and immune status; but do not capture systemic inflammation. In SA-AKI, nutritional status is closely linked to inflammatory burden, immune competence and renal recovery, suggesting that an integrated biomarker may improve risk assessment.

The C-reactive protein-albumin-lymphocyte (CALLY) index is a novel composite biomarker integrating systemic inflammation, nutritional status, and immune competence (10). Its prognostic value has been demonstrated in oncologic and cardiovascular diseases (11–15), and lower CALLY index values have also been associated with adverse outcomes in critically ill patients with sepsis (16). Because SA-AKI is characterized by concurrent inflammatory, nutritional, and immunity disturbances, the CALLY index may offer particular value for risk stratification in this population. However, its prognostic role in SA-AKI remains unclear. Therefore, this study aimed to evaluate the prognostic value of early CALLY index measurements in ICU patients with SA-AKI.

## Materials and methods

### Study design and population

This two-center retrospective cohort study was conducted at the Second Affiliated Hospital and Yuying Children’s Hospital of Wenzhou Medical University, and at Taizhou Hospital of Zhejiang Province.

Patients admitted between January 2018 and December 2023 were screened for eligibility. The study was approved by the institutional review boards of the participating centers (Approval No. 2508071523268), and the requirement for informed consent was waived because of the retrospective design and the use of anonymized data. The study was conducted in accordance with the Declaration of Helsinki and reported following the STROBE statement (17, 18).

Consecutive adult patients (≥18 years) admitted to the ICU with sepsis were screened. Sepsis was defined according to the Sepsis-3.0 criteria as life-threatening organ dysfunction caused by a dysregulated host response to infection (19). The criteria for identifying infection were as follows: (1) microbiologically confirmed infection: at least one positive result from pathogen testing; (2) clinically diagnosed infection: all pathogen tests were negative, but the attending physician made a definite diagnosis of infection based on clinical manifestations (fever/hypothermia, leukocyte changes, imaging findings, etc.) (20). The study cohort was restricted to patients who developed AKI within 7 days after sepsis onset, in accordance to KDIGO criteria (21). AKI was defined as an increase in serum creatinine of ≥0.3 mg/dL within 48 h, an increase in serum creatinine to ≥1.5 times baseline within the previous 7 days, or a urine output of <0.5 mL/kg/h for more than 6 h. As most patients had no pre-admission serum creatinine records, the first serum creatinine value measured after admission was used as the baseline. To assess the impact of baseline creatinine choice on SA-AKI diagnosis, we back-calculated an estimated pre-admission baseline creatinine using the Modification of Diet in Renal Disease (MDRD) equation (assuming eGFR 75 mL/min/1.73m^2^, adjusted for age and sex) for patients without known pre-admission creatinine or CKD. We then determined, among the original SA-AKI patients (admission creatinine-based), the proportion who still met at least one KDIGO criterion after applying the estimated baseline. The exclusion criteria were as follows: chronic kidney disease with an estimated glomerular filtration rate of <60 mL/min/1.73 m^2^ for more than three months, previous kidney transplantation, pregnancy, non-primary ICU admissions, ICU stay of less than 48 h, and missing follow-up data. After the application of these criteria, 1,019 patients were included in the final analysis.

### Data collection

Data were systematically extracted from the electronic medical record systems of the two participating centers. Diagnoses and procedures were standardized according to the International Classification of Diseases, Tenth Revision (ICD-10) (22). Variables collected within 48 h of ICU admission included demographic characteristics, comorbidities, vital signs, therapeutic interventions [including mechanical ventilation, vasopressors, and continuous renal replacement therapy (CRRT)], laboratory parameters, and illness severity scores [APACHE II (23), SAPS II (24), SOFA (25)]. The date of ICU admission was designated as Day 1. CRP, albumin, and lymphocyte counts were measured serially on Days 3, 7, and 14, and the corresponding CALLY index was calculated for each timepoint. The CALLY index was calculated using the following formula: 100 × [Albumin (g/L) × Lymphocyte count (×10^9^/L)] /CRP (mg/L).

### Outcomes

The primary outcome were all-cause mortality at 30-day, 60-day, and 365-day, as well as in-hospital mortality. Secondary outcomes included ICU length of stay and total hospital length of stay.

### Statistical analysis

Continuous variables are presented as mean ± standard deviation (SD) for normally distributed data or as median (interquartile range, IQR) for non-normally distributed data. Categorical variables are expressed as numbers and percentages. Between-group comparisons were performed using Student’s t test, the Mann–Whitney U test, or the chi-square test, as appropriate. All tests were two-tailed, and a *p* value < 0.05 was considered statistically significant.

#### Handling of missing-data

In accordance with a prespecified 20% threshold for missingness, variables with <20% missing data were handled using multiple imputation with 5 imputations. For variables with missingness ≥20% that were considered clinically essential, such as the CALLY index, imputation was also performed, followed by sensitivity analyses to assess the robustness of the results. Variables with a high degree of missingness that were not considered clinically essential were evaluated for exclusion (26). For the Day 3 CALLY index, 17 of 1,019 patients (1.7%) had missing values. Given the extremely low proportion of missingness and the comparable baseline characteristics between patients with and without missing data (Supplementary Table A2), we performed a complete-case analysis for this variable.

#### Determination of the primary exposure timepoint

To determine the primary exposure timepoint, patients were stratified into quartiles according to the Day 1, Day 3, Day 7, and Day 14 CALLY index values. Kaplan–Meier survival analysis and log-rank testing were used to compare the associations of these two timepoints with mortality. The timepoint showing the strongest prognostic discrimination was selected as the primary exposure variable for subsequent analyses.

#### Covariates were selected according to the following principles

(1) demographic characteristics; (2) variables previously reported to influence the CALLY index or the prognosis of SA-AKI; (3) variables that changed the model estimate by >10% when introduced, in accordance with STROBE recommendations (18); and (4) variables supported by clinical judgment. Based on these criteria, the covariates included were age, sex, hypertension, diabetes mellitus, chronic liver disease, CRRT, fluid resuscitation, APACHE II score, procalcitonin (PCT, ng/mL), lactate (mmol/L), gastrointestinal infection, and fungal infection.

#### Modeling of nonlinear relationships

To investigate potential nonlinear associations between the CALLY index and mortality, restricted cubic spline regression with four knots was applied. When evidence of nonlinearity was identified, inflection points were estimated using a recursive algorithm, and threshold effects were evaluated by comparing a standard linear model with two-piecewise linear model. The 95% confidence intervals for the inflection points were estimated using bootstrap resampling with 5,000 iterations.

#### Cox proportional hazards models

Univariate Cox regression analyses were first performed to identify variables associated with 30-day mortality. Multivariable Cox proportional hazards models were then used to estimate hazard ratios (HRs) and 95% confidence intervals (CIs) for the associations between the CALLY index and mortality outcomes. Covariates were selected on the basis of clinical relevance and prior literature. Model 1 was unadjusted. Model 2 was adjusted for age, gender, hypertension, and diabetes mellitus. Model 3 was further adjusted for chronic liver disease, CRRT, fluid resuscitation, APACHE II score, procalcitonin, lactate, gastrointestinal infection, and fungal infection. Subgroup analyses were conducted according stratified by age, sex, APACHE II score, CRRT use, chronic kidney disease, hypertension, diabetes, respiratory failure, and mechanical ventilation use to evaluate the robustness of the findings.

#### Mediation analysis

This exploratory analysis was performed to quantify the extent to which the CALLY index mediated the association between the age and mortality, as previously described (27). The total effect was decomposed into direct and indirect effects, with the indirect effect operating through the CALLY index. The indirect effect was estimated using bootstrap resampling with 5,000 iterations, and the proportion mediated, together with its 95% CI, was reported.

#### Bayesian joint models

Bayesian joint models with shared random effects were used to evaluate the association between longitudinal CALLY index measurements (obtained on Days 1, 3, 7, and 14) and 30-day mortality while accounting for informative dropout. These shared-parameter models combine a linear mixed-effects submodel for the longitudinal biomarker trajectory with a Cox proportional hazards submodel for survival, linking the two processes through subject-specific latent random effects after covariate adjustment (25).

To reduce potential bias related to missing data, Multiple Imputation by Chained Equations (MICE) was applied to impute missing CALLY index values at later time points (Days 7 and 14). Five imputed datasets were generated, and joint modeling analyses were performed separately in each dataset. The final pooled estimates and standard errors were combined according to Rubin’s rules.

Natural cubic splines were incorporated into both the fixed and random effects to account for potential nonlinearity in the longitudinal trajectories. Because the initial joint model assumed a time-constant association between the longitudinal CALLY index and mortality, we further extended the model by introducing an interaction term between the longitudinal CALLY index and a time-based restricted cubic spline with four knots. This approach allowed us to evaluate whether the strength of the association varied over the follow-up period.

This approach allowed us to evaluate whether the strength of the association varied over the follow-up period. To further quantify the incremental predictive value of longitudinal CALLY dynamics, we compared two nested models using the Deviance Information Criterion (DIC): Model 0 included baseline CALLY and all covariates without longitudinal dynamics, whereas Model 1 additionally incorporated longitudinal CALLY features (individual slopes, mean levels, and ranges of change). A ΔDIC < −5 was considered evidence of substantial improvement (28).

All statistical analyses were performed using R software (version 4.2.3) and SPSS (version 27).

## Results

**Figure 1:**
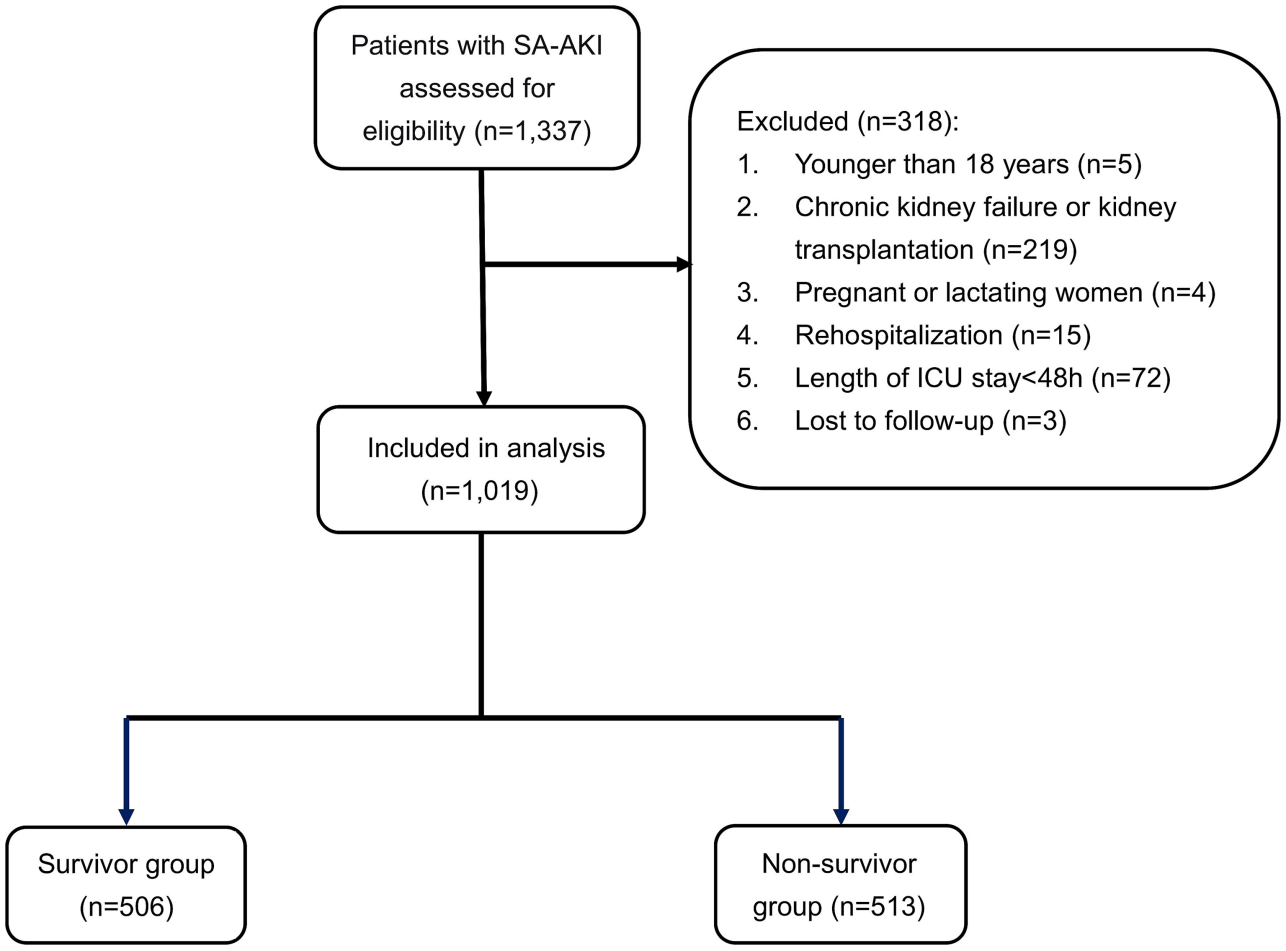
Flow diagram of patient screening, eligibility assessment, inclusion, and grouping. ICU, intensive care unit. SA-AKI, sepsis-associated acute kidney injury.

In this retrospective cohort study, 1,019 of 1,337 patients diagnosed with SA-AKI met the inclusion criteria (Figure 1). The overall mortality rates were 50.3% at 30 days, 52.7% at 60 days, 57.1% at 365 days, and 55.8% during the index hospitalization. Baseline characteristics stratified by 30-day survival status are presented in Table 1. Compared to survivors, non-survivors were older, were more frequently male, and had a higher prevalence of comorbidities, including heart failure, chronic liver disease, malignancy, and respiratory dysfunction. Non-survivors also had lower CRP levels, lower serum albumin levels, and required more intensive supportive therapies, including CRRT, vasoactive agents, fluid resuscitation, mechanical ventilation, and corticosteroids. Notably, the Day 1 CALLY index did not differ significantly between non-survivors and survivors (median 13.0 [IQR 6.1–32.9] vs. 11.7 [IQR 5.8–25.4]; *p* = 0.237), whereas the Day 3 CALLY index was significantly lower in non-survivors than in survivors (median 11.2 [IQR 5.2–25.2] vs. 16.1 [IQR 8.7–37.6]; *p* < 0.001).

**Table 1:** Baseline characteristics of the survivors and non-survivors.

Characteristics	All patients (*n* = 1,019)	Survivors (*n* = 506)	Non-survivors (*n* = 513)	*p* value
Age (years)	74 (64–82)	72 (61–81)	76 (67–83)	<0.001
BMI, kg/m^2^	21.55 (20.76–23.63)	21.62 (20.76–23.88)	21.55 (20.45–23.31)	0.080
Male, *n* (%)	670 (66%)	298 (59%)	372 (73%)	<0.001
Comorbidities, *n* (%)				
Heart failure	291 (28.56%)	116 (22.92%)	175 (34.11%)	<0.001
Atrial fibrillation	192 (18.84%)	87 (17.19%)	105 (20.47%)	0.181
Renal disease	313 (30.75%)	154 (30.43%)	159 (31.05%)	0.830
Chronic liver disease	239 (23.45%)	95 (18.77%)	144 (28.07%)	<0.001
COPD	63 (6.18%)	25 (4.94%)	38 (7.41%)	0.102
CAD	161 (15.80%)	78 (15.42%)	83 (16.18%)	0.738
Stroke	209 (20.51%)	107 (21.15%)	102 (19.88%)	0.618
Malignancy	230 (22.57%)	82 (16.21%)	148 (28.85%)	<0.001
Respiratory failure	607 (59.57%)	173 (34.19%)	434 (84.60%)	<0.001
ARDS	122 (11.97%)	34 (6.72%)	88 (17.15%)	<0.001
Pneumonia	656 (64.38%)	257 (50.79%)	399 (77.78%)	<0.001
Hypertension	449 (44.06%)	227 (44.86%)	222 (43.27%)	0.61
Diabetes mellitus	319 (31.31%)	166 (32.81%)	153 (29.82%)	0.305
Site of infection, *n* (%)				
Multiple infections	429 (42.10%)	228 (45.06%)	201 (39.18%)	<0.001
Lower respiratory tract infection	636 (62.41%)	257 (50.79%)	379 (73.88%)	<0.001
Gastrointestinal infection	52 (5.10%)	42 (8.30%)	10 (1.95%)	<0.001
Intra-abdominal infection	227 (22.28%)	89 (17.59%)	138 (26.90%)	<0.001
Urinary tract infection	254 (24.93%)	189 (37.35%)	65 (12.67%)	<0.001
Bacteremia	328 (32.19%)	189 (37.35%)	139 (27.10%)	<0.001
Skin and soft tissue infection	71 (6.97%)	37 (7.31%)	34 (6.63%)	0.668
Laboratory tests				
White blood cell count, ×10^9^/L	12.3 (7.8–18.5)	12.8 (9.0–19.4)	11.2 (6.4–17.9)	<0.001
Lymphocyte count, ×10^9^/L	0.60 (0.38–1.00)	0.60 (0.40–1.03)	0.60 (0.40–1.03)	0.234
C-reactive protein, mg/L	146.0 (87.6–212.1)	155.9 (99.7–219.0)	136.6 (75.7–201.6)	<0.001
Day 1 CALLY index	12.2 (5.9–28.7)	11.7 (5.8–25.4)	13.0 (6.1–32.9)	0.237
Day 3 CALLY index	13.9 (6.7–31.4)	16.1 (8.7–37.6)	11.2 (5.2–25.2)	<0.001
Day 7 CALLY index	42.7 (17.8–139.9)	80.1 (33.6–230.2)	19.5 (10.6–40.6)	<0.001
Day 14 CALLY index	56.8 (18.4–302.9)	145.7 (42.7–658.2)	18.6 (10.3–34.1)	<0.001
Albumin, g/L	28.90 (25.10–32.40)	29.5 (25.6–33.0)	28.3 (24.3–31.7)	<0.001
Creatinine, μmol/L	152.0 (107.4–230.7)	147.5 (112.8–211.5)	159.3 (101.0–248.0)	0.894
Lactate, mmol/L	2.5 (1.6–4.4)	2.2 (1.5–3.7)	2.9 (1.8–5.5)	<0.001
PCT, ng/mL	8.6 (2.1–38.2)	13.5 (3.0–59.6)	6.0 (1.6–25.3)	<0.001
Severity scores				
SOFA score	9 (6–11)	6 (4–9)	11 (9–13)	<0.001
APACHE II score	19 (14–24)	16 (12–20)	22 (18–27)	<0.001
SAPS II score	43 (36–54)	39 (33–47)	50 (40–63)	<0.001
Treatments, *n* (%)				
CRRT	239 (23.5%)	69 (13.6%)	170 (33.1%)	<0.001
Vasoactive agents	725 (71.1%)	297 (58.7%)	428 (83.4%)	<0.001
Fluid resuscitation	623 (61.1%)	370 (73.1%)	253 (49.3%)	<0.001
Mechanical ventilation	596 (58.5%)	146 (28.9%)	450 (87.7%)	<0.001
Corticosteroid therapy	333 (32.7%)	112 (22.1%)	221 (43.1%)	<0.001
Outcomes				
ICU length of stay, days	6 (3–13)	5 (3–12)	7 (4–14)	<0.001

*N* (%) or median (Q1-Q3) or mean ± standard deviation. ALT, alanine transaminase; APTT, activated partial thromboplastin time; BMI, body mass index; CAD, coronary artery disease; COPD, chronic obstructive pulmonary disease; CRRT, continuous renal replacement therapy; PCT, procalcitonin.

The effective sample sizes and 30-day mortality events at each CALLY measurement time point are provided in Supplementary Table A1. The number of patients with available CALLY measurements decreased from 1,019 on Day 1 to 486 on Day 14, with the greatest reduction occurring after Day 7. The corresponding 30-day mortality rates among patients with available measurements were 50.3% at Day 1, 50.3% at Day 3, 39.8% at Day 7, and 36.6% at Day 14.

Using estimated preadmission baseline creatinine in the sensitivity analysis, 878 patients (86.2%) remained eligible according to the creatinine-based relative increase criterion. Of the 141 patients (13.8%) who no longer met this criterion, 126 (89.4%) still met the urine output criterion for SA-AKI, and only 15 patients (1.5%) were reclassified as non-AKI because none of the three KDIGO criteria were met. Overall, 98.5% (1,004/1,019) of the initially identified SA-AKI patients still fulfilled at least one KDIGO criterion after applying the estimated pre-admission baseline, supporting the robustness of the SA-AKI definition. In addition, patients with missing Day 3 CALLY values were comparable to those with complete Day 3 data across baseline characteristics, with no significant differences observed (Supplementary Table A2; all *p* > 0.05).

**Figure 2:**
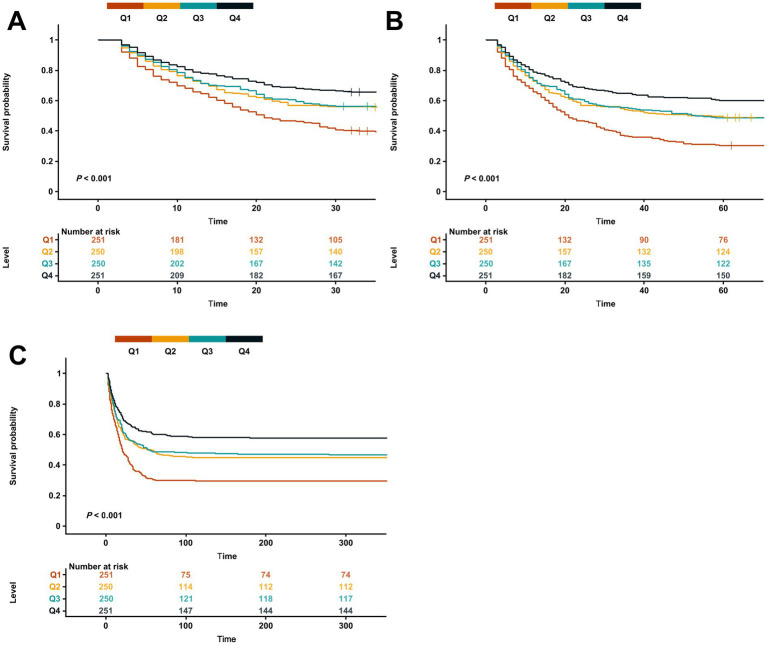
Kaplan–Meier survival curves for mortality in SA-AKI stratified by Day 3 CALLY index quartiles. **(A)** 30-day, **(B)** 60-day, **(C)** 365-day. Quartiles cutoffs were defined as follows: Q1 ≤ 6.74, Q2 6.74–13.88, Q3 13.88–30.09, and Q4 > 30.09. Survival distributions differed significantly across quartiles in all analyses (log-rank *p* < 0.001).

Kaplan–Meier survival analyses were performed to assess differences in survival across quartiles of the CALLY index on Day 1, Day 3, Day 7, and Day 14. No significant differences in survival were observed among Day 1 CALLY index quartiles (log-rank *p* > 0.05; Supplementary Figure A1). In contrast, higher quartiles of the Day 3 CALLY index were associated with a significantly lower risk of 30-day mortality (log-rank *p* < 0.0001; Figure 2), and this pattern remained consistent for 60-day, 365-day, and in-hospital mortality. Similarly, the Day 7 and Day 14 CALLY index also showed significant associations with mortality (log-rank *p* < 0.001 for both; Supplementary Figures A2, A3). Although Day 7 and Day 14 demonstrated good prognostic discrimination, Day 3 was selected as the primary exposure timepoint because it provided the earliest risk stratification while maintaining sufficient sample size and clinical relevance. Delaying risk assessment to Day 7 or Day 14 would miss the critical early therapeutic window for intervention.

Table 2 presents the clinical characteristics and outcomes of patients stratified by quartiles of the Day 3 CALLY index. Due to partial missing data in this index (*n* = 17), the analysis included 1,002 patients. Patients in the higher CALLY index quartiles had lower disease severity scores, lower procalcitonin and lactate levels, and less frequent use of vasoactive agents and mechanical ventilation. Intra-abdominal and urinary tract infections were less common in these groups. In addition, patients with higher CALLY index values had shorter ICU stays and lower 30-day mortality, with similar trends observed for 60-day and 365-day mortality.

**Table 2:** Clinical characteristics and outcomes of patients with SA-AKI stratified by quartiles of the Day 3 CALLY index.

Characteristics	Q1 ≤ 6.74 (*n* = 251)	Q2 6.74–13.88 (*n* = 250)	Q3 13.88–30.09 (*n* = 251)	Q4 > 30.09 (*n* = 250)	*p* value
Age (years)	73 (65–83)	76 (66–83)	74 (66–83)	72 (61–80)	0.040
BMI, kg/m^2^	21.51 (20.45–23.31)	22.04 (20.76–23.91)	21.55 (20.76–23.31)	21.55 (20.81–23.67)	0.290
Male, *n* (%)	182 (73%)	168 (67%)	160 (64%)	147 (59%)	0.010
Comorbidities, *n* (%)					
Heart failure	76 (30.28%)	81 (32.40%)	69 (27.60%)	59 (23.51%)	0.143
Atrial fibrillation	47 (18.73%)	53 (21.20%)	47 (18.80%)	39 (15.54%)	0.443
Renal disease	79 (31.47%)	82 (32.80%)	75 (30.12%)	74 (29.48%)	0.858
Chronic liver disease	43 (17.13%)	56 (22.40%)	70 (28.00%)	65 (25.90%)	0.024
COPD	18 (7.17%)	15 (6.00%)	12 (4.80%)	16 (6.37%)	0.734
CAD	32 (12.75%)	32 (12.80%)	48 (19.20%)	42 (16.73%)	0.123
Stroke	39 (15.54%)	60 (24.00%)	47 (18.80%)	60 (23.90%)	0.049
Malignancy	79 (31.47%)	48 (19.20%)	53 (21.20%)	46 (18.33%)	0.001
Respiratory failure	177 (70.52%)	148 (59.20%)	143 (57.20%)	130 (51.79%)	<0.001
ARDS	33 (13.15%)	35 (14.00%)	28 (11.20%)	22 (8.76%)	0.271
Pneumonia	175 (69.72%)	161 (64.40%)	163 (65.20%)	150 (59.76%)	0.139
Hypertension	113 (45.02%)	109 (43.60%)	100 (40.00%)	121 (48.21%)	0.317
Diabetes mellitus	77 (30.68%)	85 (34.00%)	70 (28.00%)	82 (32.67%)	0.498
Site of infection, *n* (%)					
Multiple infections	114 (45.42%)	114 (45.60%)	105 (42.00%)	94 (37.45%)	0.213
Lower respiratory tract infection	171 (68.13%)	154 (61.60%)	156 (62.40%)	149 (59.36%)	0.213
Gastrointestinal infection	9 (3.59%)	8 (3.20%)	17 (6.80%)	17 (6.77%)	0.111
Intra-abdominal infection	75 (29.88%)	62 (24.80%)	51 (20.40%)	38 (15.14%)	<0.001
Urinary tract infection	40 (15.94%)	79 (31.60%)	56 (22.40%)	75 (29.88%)	<0.001
Bacteremia	88 (35.06%)	74 (29.60%)	83 (33.20%)	77 (30.68%)	0.553
Skin and soft tissue infection	20 (7.97%)	14 (5.60%)	19 (7.60%)	17 (6.77%)	0.735
Laboratory tests					
White blood cell count, ×10^9^/L	8.8 (4.7–15.3)	12.3 (8.9–19.1)	13.1 (8.9–19.8)	13.7 (9.5–19.2)	<0.001
Lymphocyte count, ×10^9^/L	0.40 (0.22–0.60)	0.55 (0.40–0.90)	0.65 (0.41–1.05)	0.91 (0.51–1.37)	<0.001
C-reactive protein, mg/L	191.1 (126.0–259.9)	162.2 (107.9–219.7)	142.3 (88.3–188.6)	98.4 (53.3–156.0)	<0.001
Albumin, g/L	27.5 (23.6–30.7)	27.9 (24.1–31.3)	29.6 (25.3–33.5)	30.1 (27.1–33.7)	<0.001
Creatinine, μmol/L	183.8 (114.4–257.7)	157.5 (110.0–237.2)	138.3 (101.3–205.8)	138.0 (100.8–208.5)	<0.001
Lactate, mmol/L	3.0 (1.9–5.1)	2.7 (1.8–4.7)	2.5 (1.6–4.5)	2.0 (1.3–3.2)	<0.001
PCT, ng/mL	19.0 (5.2–64.3)	10.7 (2.6–48.2)	7.6 (1.5–33.4)	4.2 (0.8–17.2)	<0.001
Severity scores					<0.001
SOFA score	10 (8–12)	9 (6–12)	9 (6–11)	7 (5–10)	<0.001
APACHE II score	21 (18–26)	18 (15–22)	18 (14–24)	17 (13–22)	<0.001
SAPS II score	49 (38–58)	44 (36–56)	43 (36–54)	40 (33–49)	<0.001
Treatments, *n* (%)					
CRRT	71 (28.29%)	66 (26.40%)	66 (26.40%)	53 (21.12%)	0.054
Vasoactive agents	218 (86.85%)	191 (76.40%)	164 (65.60%)	141 (56.18%)	<0.001
Fluid resuscitation	160 (63.75%)	166 (66.40%)	150 (60.00%)	138 (54.98%)	0.051
Mechanical ventilation	182 (72.51%)	146 (58.40%)	140 (56.00%)	119 (47.41%)	<0.001
Corticosteroid therapy	106 (42.23%)	82 (32.80%)	75 (30.00%)	67 (26.69%)	0.002
Outcomes					
ICU length of stay, days	8 (4–15)	7 (4–14)	6 (3–11)	5 (2–10)	<0.001
Hospital length of stay, days	14 (7–22)	14 (9–23)	13 (8–22)	12 (8–21)	0.414
30-day mortality	168 (66.93%)	118 (47.20%)	120 (48.00%)	98 (39.04%)	<0.001
60-day mortality	175 (69.72%)	128 (51.20%)	128 (51.20%)	101 (40.24%)	<0.001
365-day mortality	188 (74.90%)	139 (55.60%)	135 (54.00%)	111 (44.22%)	<0.001
In-hospital mortality	184 (73.31%)	135 (54.00%)	131 (52.40%)	104 (41.43%)	<0.001

*N* (%) or median (Q1-Q3) or mean ± standard deviation. ALT, alanine transaminase; APTT, activated partial thromboplastin time; BMI, body mass index; CAD, coronary artery disease; COPD, chronic obstructive pulmonary disease; CRRT, continuous renal replacement therapy; PCT, procalcitonin. Due to partial missing data in the Day 3 CALLY index (*n* = 17 missing), the total sample size for this analysis is 1,002.

**Figure 3:**
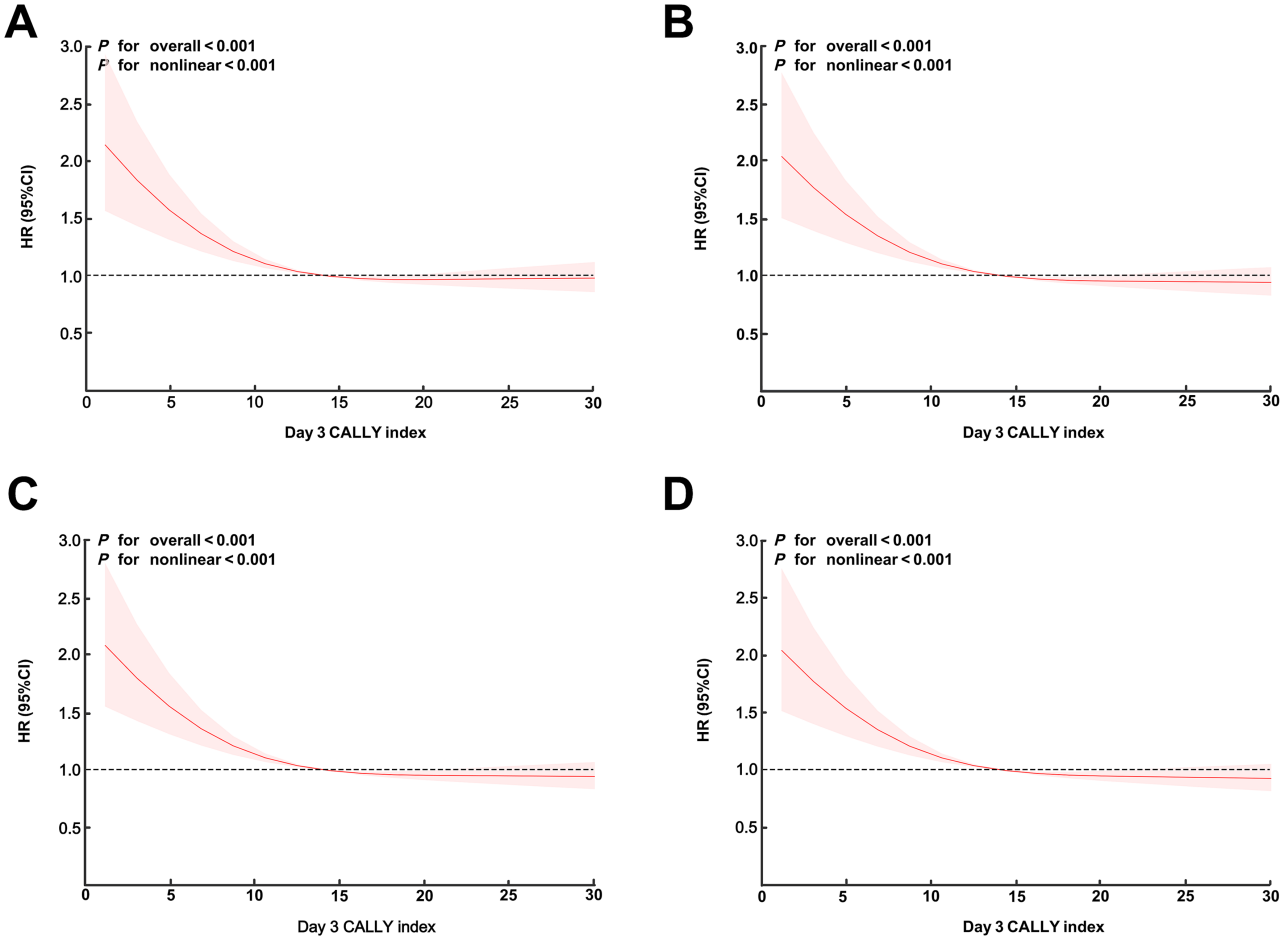
Restricted cubic spline analysis of the association between the Day 3 CALLY index and mortality in patients with SA-AKI. **(A)** 30-day mortality, **(B)** 60-day mortality, **(C)** 365-day mortality, and **(D)** in-hospital mortality. The solid red line indicates the hazard ratio, and the pink shaded area represents the 95% confidence interval (CI).

Restricted cubic spline analysis revealed a nonlinear, L-shaped association between the Day 3 CALLY index and mortality risk in patients with SA-AKI (Figures 3A–D). Mortality risk declined sharply at lower CALLY index values and then gradually plateaued beyond a threshold. This nonlinear relationship was further supported by threshold effect analysis (Table 3; Supplementary Table A3). For 30-day mortality, an exploratory threshold was observed at a Day 3 CALLY index of 11.52 (95% CI: 9.98–14.73). Below this threshold, each one-unit increase in the CALLY index was associated with a 10.6% lower mortality risk (hazard ratio [HR] 0.894, 95% CI 0.824–0.970, *p* = 0.007). Above this threshold, the protective association was significantly attenuated, with only a 0.3% reduction in risk per unit increase (HR 0.997, 95% CI 0.994–1.000, *p* = 0.055). Similar L-shaped associations were observed for 60-day, 365-day, and in-hospital mortality, with inflection points at 12.63 (95% CI: 9.31–14.02), 12.65 (95% CI: 9.70–14.32), and 13.56 (95% CI: 10.20–14.83), respectively. Log-likelihood ratio tests demonstrated that the two-piecewise linear models provided a significantly better fit than the conventional linear models for all outcomes (all *p* < 0.001).

**Table 3:** Threshold effect of Day 3 CALLY index on 30-day mortality in patients with SA-AKI.

Model	HR (95% CI)	*p* value
Model I		
Linear-effect model	0.996 (0.993–0.999)	0.004
Model II		
Inflection point(K)	11.52 (9.98–14.73)	
< K	0.894 (0.824–0.970)	0.007
> K	0.997 (0.994–1.000)	0.055
Log likelihood ratio	<0.001	

CI, confidence interval; HR, hazard ratio.

In univariate Cox proportional hazards analyses, higher CALLY index values on Days 3, 7, and 14 were each significantly associated with lower 30-day mortality (all *p* < 0.001, HR < 1, Supplementary Table A4). Multivariable Cox regression further confirmed the inverse association between the Day 3 CALLY index and mortality risk; specifically, each one-unit increase in the Day 3 CALLY index was associated with a lower hazard for 30-day mortality (HR 0.997, 95% CI 0.995–0.999, *p* = 0.008; Table 4). In the fully adjusted Model 3, patients in the highest quartile of the Day 3 CALLY index had significantly lower mortality hazards than those in the lowest quartile, with HRs of 0.631 (95% CI 0.483–0.824) for 30 days mortality, 0.589 (95% CI 0.453–0.764) for 60 days mortality, 0.587 (95% CI 0.456–0.754) for 365 days mortality, and 0.569 (95% CI 0.440–0.736) for in-hospital mortality (Supplementary Table A5).

**Table 4:** Multivariable cox regression of the association between the Day 3 CALLY index and 30-day mortality in patients with SA-AKI.

Variable	Model 1		Model 2		Model 3	
	HR (95% CI)	*p* value	HR (95% CI)	*p* value	HR (95% CI)	*p* value
Continuous variable	0.996 (0.994–0.999)	0.001	0.997 (0.995–0.999)	0.004	0.997 (0.995–0.999)	0.008
Quartile						
Q1	1		1		1	
Q2	0.620 (0.490–0.785)	<0.001	0.627 (0.496–0.794)	<0.001	0.811 (0.636–1.033)	0.089
Q3	0.615 (0.487–0.778)	<0.001	0.624 (0.493–0.790)	<0.001	0.669 (0.525–0.853)	<0.001
Q4	0.468 (0.365–0.601)	<0.001	0.510 (0.396–0.655)	<0.001	0.631 (0.483–0.824)	<0.001
*P* for trend	0.004		0.003		0.007	

Model 1: unadjusted. Model 2: adjusted for: sex, age, hypertension, diabetes mellitus. Model 3: fully adjusted (Model 2 + chronic liver disease, CRRT, fluid resuscitation, APACHE II score, procalcitonin, lactate, gastrointestinal infection, fungal infection).

**Figure 4:**
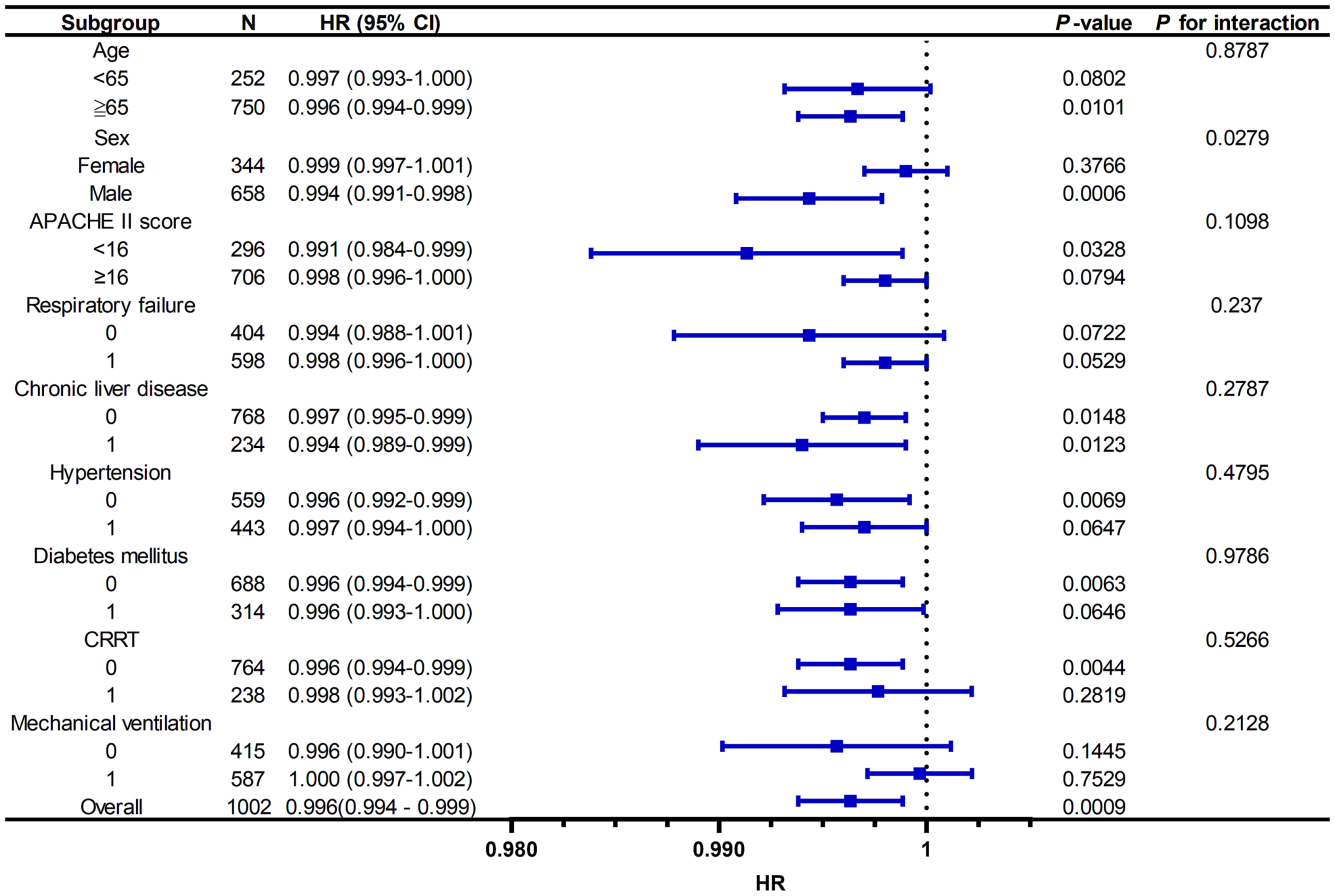
Forest plot of subgroup analyses for the association between the Day 3 CALLY index and 30-day mortality in patients with SA-AKI. Hazard ratios (HRs) with 95% confidence intervals (CIs) are shown for each subgroup, together with *p* values for interaction.

Stratified analyses were performed to assess the robustness of the association between the Day 3 CALLY index and 30-day mortality across predefined subgroups, including age, sex, APACHE II score, lactate levels, hypertension, diabetes, chronic liver disease, and the use of mechanical ventilation or CRRT. As illustrated in Figure 4, a higher Day 3 CALLY index was associated with lower 30-day mortality in several subgroups, including patients aged ≥65 years [HR 0.996, 95% CI 0.994–0.999], those without hypertension (HR 0.996, 95% CI 0.992–0.999) and in those without diabetes (HR 0.996, 95% CI 0.994–0.999). In the APACHE II-stratified analysis, patients with a score <16 (n = 296, 29.5%) showed an HR of 0.991 (95% CI 0.984–0.999, *p* = 0.0328), while those with a score ≥16 had an HR of 0.998 (95% CI 0.996–1.000, *p* = 0.0794); the interaction *p* value was 0.1098. No significant interactions were detected across the subgroups (all *P* for interaction > 0.05).

**Figure 5:**
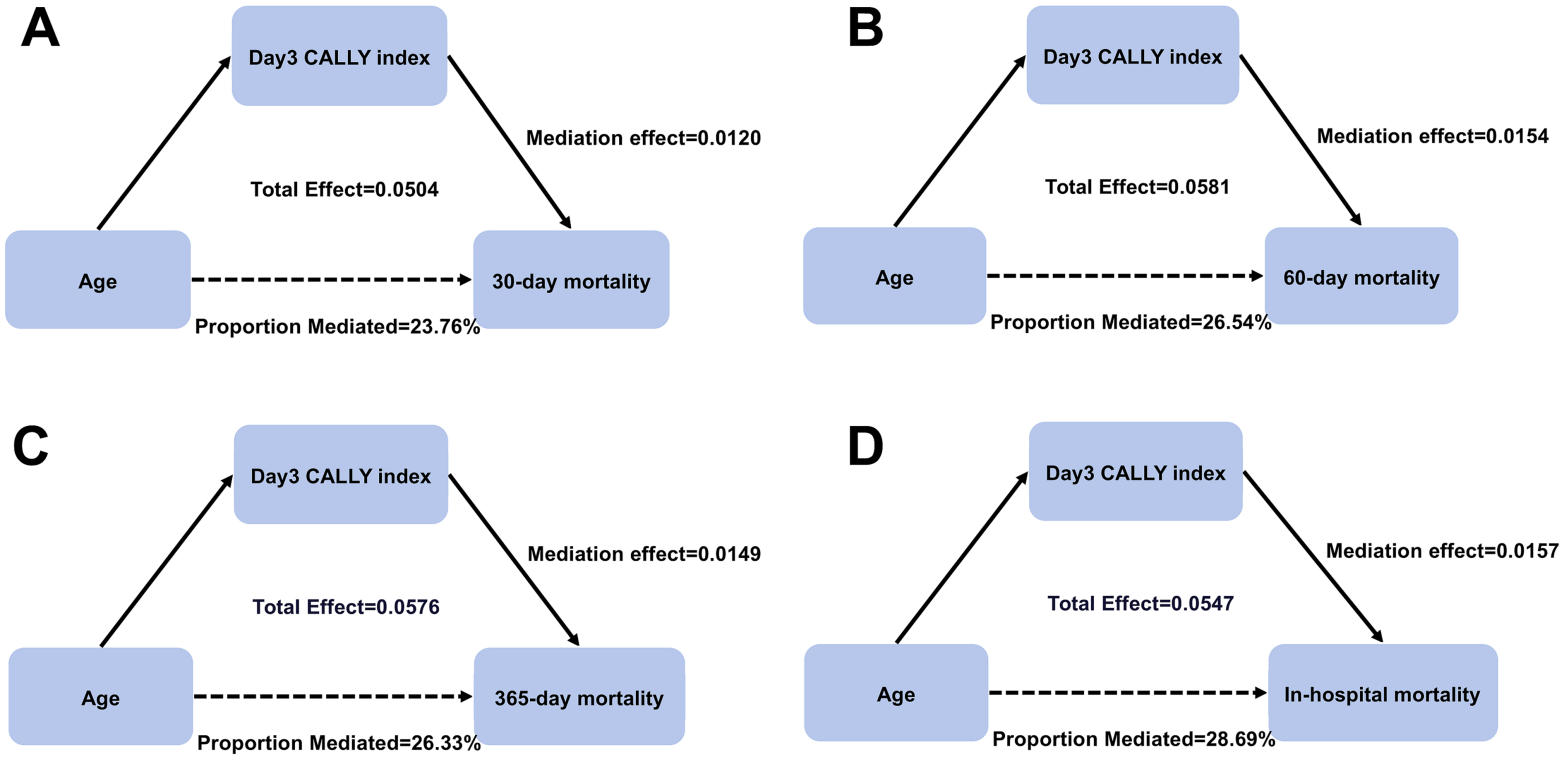
Mediation analysis of the Day 3 CALLY index in the association between age and mortality in patients with SA-AKI. **(A)** 30-day mortality, **(B)** 60-day mortality, **(C)** 365-day mortality, and **(D)** in-hospital mortality. The diagrams show the total effect of age on mortality, the indirect (mediated) effect through the Day 3 CALLY index, and the proportion mediated.

Because older age is a well-established risk factor for mortality in SA-AKI and was inversely associated with the Day 3 CALLY index in our cohort, we further examined whether the CALLY index mediated the association between age and mortality. As shown in Figure 5, age was treated as the independent variable, the Day3 CALLY index as the mediator, and mortality at 30 days, 60 days, 365 days, and during hospitalization as the dependent outcomes. Exploratory mediation analysis suggested that the Day 3 CALLY index may play a partial mediating role in the association between age and mortality. In the fully adjusted models, the proportion of the total effect of age on mortality that was statistically explained through the Day 3 CALLY index was 23.76% for 30-day mortality, 26.54% for 60-day mortality, 26.33% for 365-day mortality, and 28.69% for in-hospital mortality. The interaction term between age and the CALLY index was not statistically significant across all mortality outcomes (all *p* > 0.05), as detailed in Supplementary Table A6.

**Figure 6:**
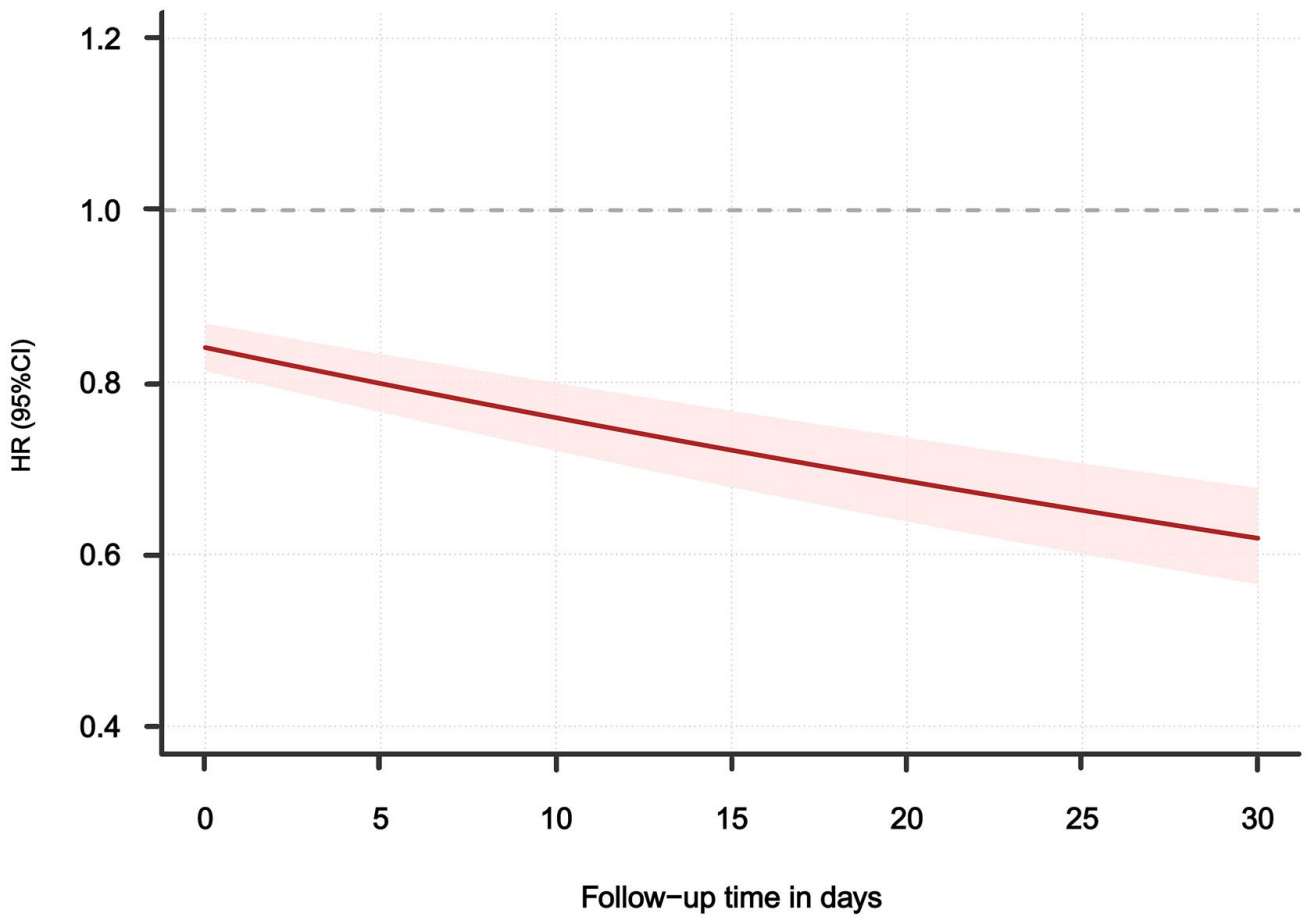
Time-varying association between the longitudinal log-transformed CALLY index and 30-day mortality estimated using a Bayesian joint model. The hazard ratio (HR) represents the effect of a one-unit increase in the current log-transformed CALLY index over follow-up. The solid red line indicates the estimated HR, and the shaded area represents the 95% credible interval.

Bayesian joint models incorporating longitudinal CALLY index measurements on Days 1, 3, 7, and 14 showed a time-dependent protective association with 30-day mortality, which became progressively stronger over follow-up (Figure 6). Model convergence and sampling efficiency were confirmed by MCMC diagnostics (all Rhat = 1.00; Bulk_ESS and Tail_ESS > 2,500 for all parameters, well above the conventional threshold of 400, confirming stable and precise estimates). After multiple imputation of missing values using MICE with five imputed datasets, the Bayesian joint model showed that the time-varying CALLY index remained significantly associated with 30-day mortality risk after adjustment for relevant covariates (HR 0.934, 95% CI 0.923–0.946; Table 5). To evaluate the stability of these findings, a complete-case sensitivity analysis was performed and the results are presented in a Supplementary Table A7. The prognostic associations were consistent with the primary MICE-based analysis, supporting the robustness of the findings.

**Table 5:** Association between time-varying CALLY index trajectories and 30-day mortality in patients with SA-AKI.

Variable	HR (95% CI)	*p* value
Age, years	1.007 (1.000–1.014)	0.0453
Sex	1.266 (1.046–1.512)	0.0155
Hypertension	0.953 (0.793–1.146)	0.6090
Diabetes mellitus	0.837 (0.689–1.018)	0.0752
Chronic liver disease	1.018 (0.831–1.247)	0.8646
CRRT	1.238 (1.026–1.495)	0.0259
Fluid resuscitation	0.694 (0.578–0.833)	<0.001
APACHE II score	1.072 (1.060–1.084)	<0.001
Procalcitonin	0.994 (0.991–0.997)	<0.001
Lactate	1.047 (1.028–1.068)	<0.001
Gastrointestinal infection	0.506 (0.297–0.865)	0.0127
Fungal infection	0.743 (0.615–0.987)	0.0021
Time-varying variables		
CALLY index	0.934 (0.923–0.946)	<0.001

CI, confidence interval; CRRT, continuous renal replacement therapy; APACHE II, Acute Physiology and Chronic Health Evaluation II; HR, hazard ratio; SA-AKI, sepsis-associated acute kidney injury. The Bayesian joint model was fitted following multiple imputation of missing values using MICE (five imputed datasets).

The model incorporating longitudinal CALLY dynamics (Model 1) yielded a substantially lower DIC (6052.66) compared with the model using only baseline CALLY (Model 0, 6508.63), with a ΔDIC of −455.96 (far below the conventional threshold of −5), indicating that the inclusion of longitudinal CALLY features significantly improved model fit (Supplementary Table A8).

## Discussion

The selection of the Day 3 CALLY index as the primary exposure variable was based on its ability to capture a critical early pathophysiological window by integrating the distinct temporal trajectories of its three component biomarkers.

CRP, with a half-life of approximately 19 h, responds rapidly to changes in systemic inflammation and therapeutic interventions. Accordingly, CRP levels on Day 1 mainly reflect the severity of the initial insult, whereas Day 3 levels more accurately indicate the host’s response to early resuscitation and antimicrobial treatment. Persistently elevated CRP at this stage has been associated with treatment failure and poor outcomes (29, 30). Serum albumin, a negative acute-phase reactant, typically reaches its nadir around Days 2–3, making Day 3 optimal for assessing nutritional and metabolic reserve depletion (31). Lymphocyte counts decline most prominently between Days 1 and 3, and Day 3 lymphopenia better reflects ongoing immune exhaustion, discriminating survivors from non-survivors (32, 33). Day 3 thus enables joint assessment of inflammatory activity, nutritional reserve, and immune competence, providing an integrated snapshot of the post-resuscitation inflammation-nutrition-immunity axis.

Although single markers such as CRP, albumin, and lymphocyte count have each been associated with mortality risk in sepsis, their predictive value as standalone markers remains limited (34–36). The CALLY index, by integrating these three components, has shown prognostic value in SA-AKI. Composite inflammatory indices derived from routine laboratory tests have attracted increasing interest because of their clinical availability. A recent study used restricted cubic splines and threshold analysis to evaluate the neutrophil counts/the prognostic nutritional index ratio in sepsis and reported similar J-shaped associations with mortality (37), which is methodologically similar to our approach. Compared with indices that primarily capture immunoinflammatory imbalance, such as the Neutrophil-to-Lymphocyte Ratio (38), and the Systemic Immune-inflammation Index (39), or ratio-based markers reflecting acute injury, such as the Lactate Dehydrogenase-to-Albumin Ratio (40), the CALLY index additionally incorporates serum albumin, enabling simultaneous evaluation of inflammatory burden, immune competence, and nutritional reserve. Consistent with this rationale, our findings showed that the CALLY index had significant prognostic value for both short- and long-term mortality in patients with SA-AKI. In our cohort, patients with lower CALLY index values were generally older and had a higher prevalence of malignancy, suggesting a vulnerable subgroup characterized by increased nutritional burden, persistent inflammation, and impaired immune competence.

The prognostic importance of dynamic biomarker assessment, rather than reliance on a single static measurement, has been increasingly recognized (41–43). Consistent with this framework, our longitudinal analysis revealed that the protective association between the CALLY index and survival became progressively stronger over time. These findings support the potential clinical utility of serial CALLY index monitoring for ongoing risk reassessment and treatment guidance in patients with SA-AKI. Notably, dynamic biomarker studies often face the challenge of missing later time points. In this regard, Xu et al. applied growth mixture modeling to identify longitudinal biomarker trajectories in burn sepsis without requiring imputation, offering a potentially more robust approach when missingness may be informative (44).

Although the CALLY index has been investigated in oncology and cardiovascular diseases (14, 45), its prognostic value in SA-AKI requires dedicated evaluation. SA-AKI is a distinct clinical entity with unique pathophysiology, differing from non-septic AKI in mechanisms, clinical phenotypes, and outcomes (46–48). Renal dysfunction may alter the clearance of inflammatory mediators, fluid imbalance may confound albumin interpretation, and the injured kidney may amplify systemic inflammation (49). These factors may fundamentally influence the behavior and clinical interpretation of the CALLY index in this population. Previous studies in general sepsis cohorts have not adequately addressed these SA-AKI specific considerations (16, 50, 51).

This study provides a comprehensive evaluation of the CALLY index for both short- and long-term mortality in SA-AKI. A key was the identification of a nonlinear, L-shaped association between Day 3 CALLY index and mortality risk. Below a distinct threshold, mortality increased sharply as the CALLY index declined, whereas above this threshold the risk plateaued, suggesting limited additional benefit from further increases. This threshold may help identify patients requiring closer monitoring and more comprehensive supportive care. In addition, an exploratory mediation analysis suggested that the Day 3 CALLY index may play a partial role in explaining the association between age and mortality, which is consistent with the concepts of inflammaging and immunosenescence (52). However, given the observational design and cross-sectional measurement of the mediator, these findings are hypothesis-generating; formal causal inference requires validation using longitudinal mediation or Mendelian randomization designs (53).

## Limitations

This study has several limitations. Firstly, as with all observational studies, residual confounding cannot be completely excluded despite comprehensive multivariable adjustments. Secondly, the data were obtained from two centers within the same geographic region, which may limit generalizability. Therefore, external validation in larger, multicenter cohorts is warranted. Thirdly, CALLY index measurements decreased after Day 3, and the widening confidence intervals after Day 7 warrant cautious interpretation. MICE (five imputed datasets) and complete-case sensitivity analysis yielded consistent results, suggesting that reduced later measurements did not materially affect our conclusions. However, when missingness is informative, imputation may still introduce bias. Fourthly, pre-admission creatinine was unavailable, which limited our ability to identify acute-on-chronic kidney injury. A sensitivity analysis using an estimated baseline (MDRD back-calculation, assuming eGFR 75) reclassified only 1.5% of patients, suggesting this limitation had minimal impact on our AKI classification.

## Conclusion

In summary, the Day 3 CALLY index was an independent prognostic marker in patients with SA-AKI. By integrating nutritional status with inflammatory and immune responses, it provided both clinically relevant early risk stratification and dynamic prognostic information over time. The identified L-shaped association with mortality further supports its potential value for the early identification of high-risk patients with impaired nutritional and metabolic reserve and for individualized management in SA-AKI.

## Data Availability

The raw data supporting the conclusions of this article will be made available by the authors, without undue reservation.

## Glossary

CALLY: C-reactive protein-albumin-lymphocyte index
SA-AKI: Sepsis-associated acute kidney injury
ICU: Intensive care unit
HR: Hazard ratio
CI: Confidence interval
IQR: Interquartile range
SD: Standard deviation
BMI: Body mass index
COPD: Chronic obstructive pulmonary disease
CAD: Coronary artery disease
ARDS: Acute respiratory distress syndrome
PCT: Procalcitonin
SOFA: Sequential organ failure assessment
APACHE II: Acute physiology and chronic health evaluation II
SAPS II: Simplified acute physiology score II
CRRT: Continuous renal replacement therapy
PaCO₂: Arterial partial pressure of carbon dioxide
BE: Base excess
HCO₃-: Bicarbonate
WBC: White blood cell
Hb: Hemoglobin
Plt: Platelet
PT: Prothrombin time
INR: International normalized ratio
APTT: Activated partial thromboplastin time
FEU: Fibrinogen equivalent units
TG: Triglycerides
ALT: Alanine aminotransferase
TBil: Total bilirubin
BUN: Blood urea nitrogen
MDRD: Modification of Diet in Renal Disease
