# Supplementary material for: Baseline and dynamic CALLY index changes predict mortality in sepsis-associated acute kidney injury: a two-center retrospective cohort study
Source: Front Nutr. 2026 Jul 28;13:1858222. doi: 10.3389/fnut.2026.1858222 (PMC13457148; doi:10.3389/fnut.2026.1858222)
Supplement: Supplementary file 1 [file Table_1.docx]

**Table A1.Effective patient numbers, missing data, and 30-day mortality events at each CALLY measurement time point**

| **Variable** | **Effective Patients, （n）** | **Missing,**  **（n）** | **30-Day mortality,**  **n (%)** |
| --- | --- | --- | --- |
| **Day 1** | 1019 | 0 | 513（50.3） |
| **Day 3** | 1002 | 17 | 504（50.3） |
| **Day 7** | 774 | 245 | 308（39.8） |
| **Day 14** | 486 | 533 | 178（36.6） |

**Notes:** CALLY, C-reactive protein/albumin-lymphocyte index; Effective patients were defined as those with available CALLY measurements at the corresponding time point.

**Table A2. Comparison of baseline characteristics between patients with and without missing Day 3 CALLY index values**

| **Characteristics** | **All patients**  **(n = 1019)** | ****Missing group****  ****(n=**17**)**** | ****Complete group****  ****(n=**1012**)**** | ***P* *value*** |
| --- | --- | --- | --- | --- |
| **Age (years)** | 74 (64–82) | 72 (66–80) | 74 (64–82) | 0.781 |
| **BMI, kg/m²** | 21.55  (20.76–23.63) | 21.50  (21.16–22.38) | 21.55  (20.76–23.63) | 0.548 |
| **Male, n (%)** | 670 (66%) | 13 (76.47%) | 657 (65.63%) | 0.350 |
| ****Comorbidities,n (%)**** |  |  |  |  |
| **Heart failure** | 291 (28.56%) | 6 (35.29%) | 285 (28.44%) | 0.535 |
| **Atrial fibrillation** | 192 (18.84%) | 6 (35.29%) | 186 (18.56%) | 0.080 |
| **Renal disease** | 313 (30.75%) | 3 (17.65%) | 310 (30.97%) | 0.238 |
| **Chronic liver disease** | 239 (23.45%) | 5 (29.41%) | 234 (23.35%) | 0.559 |
| **COPD** | 63 (6.18%) | 2 (11.76%) | 61 (6.09%) | 0.335 |
| **CAD** | 161 (15.80%) | 5 (29.41%) | 156 (15.57%) | 0.121 |
| **Stroke** | 209 (20.51%) | 3 (17.65%) | 206 (20.56%) | 0.768 |
| **Malignancy** | 230 (22.57%) | 4 (23.53%) | 226 (22.55%) | 0.924 |
| **Respiratory failure** | 607 (59.57%) | 9 (52.94%) | 598 (59.68%) | 0.574 |
| **ARDS** | 122 (11.97%) | 4 (23.53%) | 118 (11.78%) | 0.139 |
| **Pneumonia** | 656 (64.38%) | 8 (47.06%) | 648 (64.67%) | 0.133 |
| **Hypertension** | 449 (44.06%) | 6 (35.29%) | 443 (44.21%) | 0.463 |
| **Diabetes mellitus** | 319 (31.31%) | 5 (29.41%) | 314 (31.34%) | 0.865 |
| **Site of infection, n (%)** |  |  |  |  |
| **Multiple infections** | 429 (42.10%) | 4 (23.53%) | 425 (42.42%) | 0.118 |
| **Lower respiratory tract infection** | 636  (62.41%) | 7  (41.18%) | 629  (62.77%) | 0.068 |
| **Gastrointestinal infection** | 52  (5.10%) | 1  (5.88%) | 51  (5.09%) | 0.883 |
| **Intra-abdominal infection** | 227  (22.28%) | 1  (5.88%) | 226  (22.55%) | 0.101 |
| **Urinary tract infection** | 254 (24.93%) | 4 (23.53%) | 250 (24.95%) | 0.893 |
| **Bacteremia** | 328 (32.19%) | 6 (35.29%) | 322 (32.14%) | 0.782 |
| **Skin and soft tissue infection** | 71  (6.97%) | 1  (5.88%) | 70  (6.99%) | 0.859 |
| ****Laboratory tests**** |  |  |  |  |
| **White blood cell count, ×10⁹/L** | 12.3  (7.8–18.5) | 10.5  (4.0–15.9) | 12.3  (7.9–18.6) | 0.320 |
| **Lymphocyte count, ×10⁹/L** | 0.60  (0.38–1.00) | 0.8  (0.3–1.2) | 0.6  (0.4–1.0) | 0.996 |
| **C-reactive protein, mg/L** | 146.0  (87.6–212.1) | 144.6  (118.6–195.1) | 146.1  (86.4–212.2) | 0.980 |
| **Albumin, g/L** | 27.5  (23.6–30.7) | 30.0  (27.5–38.3) | 28.9  (25.0–32.4) | 0.062 |
| **Creatinine, μmol/L** | 183.8  (114.4–257.7) | 123.0  (106.3–196.0) | 152.0  (107.8–232.9) | 0.298 |
| **Lactate, mmol/L** | 3.0 (1.9–5.1) | 3.3 (2.0–5.0) | 2.5 (1.6–4.4) | 0.300 |
| **PCT, ng/mL** | 19.0(5.2–64.3) | 3.8 (1.0–17.8) | 8.7 (2.1–38.7) | 0.199 |
| ****Severity scores**** |  |  |  |  |
| **SOFA score** | 10 (8–12) | 9 (4–14) | 9 (6–11) | 0.768 |
| **APACHE II score** | 21 (18–26) | 18 (16–21) | 19 (14–24) | 0.944 |
| **SAPS II score** | 49 (38–58) | 37 (28–56) | 43 (36–54) | 0.578 |
| ****Treatments, n (%)**** |  |  |  |  |
| **CRRT** | 71 (28.29%) | 1 (5.88%) | 238 (23.75%) | 0.085 |
| **Vasoactive agents** | 218 (86.85%) | 11 (64.71%) | 714 (71.26%) | 0.554 |
| **Fluid resuscitation** | 160 (63.75%) | 9 (52.94%) | 614 (61.28%) | 0.484 |
| **Mechanical ventilation** | 182 (72.51%) | 9 (52.94%) | 587 (58.58%) | 0.640 |
| **Corticosteroid therapy** | 106 (42.23%) | 3 (17.65%) | 330 (32.93%) | 0.183 |
| ****Outcomes**** |  |  |  |  |
| **ICU length of stay, days** | 6 (3–13) | 2 (1–4) | 6 (3–4) | 0.095 |
| **Hospital length of stay, days** | 13 (8–22) | 10 (7–15) | 13 (8–22) | 0.177 |
| **30-day mortality** | 513 (50.34%) | 9 (52.94%) | 504 (50.30%) | 0.829 |
| **60-day mortality** | 538 (52.80%) | 6 (35.29%) | 532 (53.09%) | 0.145 |
| **365-day mortality** | 582 (57.11%) | 9 (52.94%) | 573 (57.19%) | 0.726 |
| **In-hospital mortality** | 563 (55.25%) | 9 (52.94%) | 554 (55.29%) | 0.847 |

**Notes:** The missing group was defined as patients with missing Day 3 CALLY index values, and the complete group as those with complete Day 3 CALLY index data.

**Data:** N (%) or median (Q1–Q3) or mean ± standard deviation

**Abbreviations:** ALT, alanine transaminase; APTT, activated partial thromboplastin time; BMI, body mass index; CAD, coronary artery disease; COPD, chronic obstructive pulmonary disease; CRRT, continuous renal replacement therapy; PCT, procalcitonin.

**Table A3. **Threshold effect of Day 3 CALLY on 60-day, 365-day, and in-hospital mortality****

|  | **60-day mortality** | ***P* value** | **365-day mortality** | ***P* value** | **In-hospital mortality** | ***P* value** |
| --- | --- | --- | --- | --- | --- | --- |
| **Model I** |  |  |  |  |  |  |
| ****Linear-****  ****effect model**** | 0.994  (0.990–0.997) | <0.001 | 0.994  (0.990–0.997) | <0.001 | 0.993  (0.990–0.996) | <0.001 |
| **Model II** |  |  |  |  |  |  |
| **Inflection point(K)** | 12.63  (9.31–14.02) |  | 12.65  (9.70–14.32) |  | 13.56  (10.20–14.83) |  |
| **< K** | 0.881  (0.815–0.952) | 0.001 | 0.862  (0.794–0.936) | <0.001 | 0.873  (0.812–0.938) | <0.001 |
| **> K** | 0.996  (0.992–0.999) | 0.012 | 0.996  (0.993–0.999) | 0.020 | 0.995  (0.992–0.999) | 0.11 |
| **Log likelihood ratio** | <0.001 | | <0.001 | | <0.001 | |

**Notes:** **Abbreviations:** CI, confidence interval; HR, hazard ratio.

**Table A4. Univariate Cox regression analysis of factors associated with 30-day mortality in SA-AKI**

| ****Variable**** | ****HR (95% CI)**** | **P**-value**** |
| --- | --- | --- |
| Day 1 CALLY index | 1.001 (0.999 , 1.002) | 0.376 |
| Day 3 CALLY index | 0.996 (0.994 , 0.999) | 0.001 |
| Day 7 CALLY index | 0.991 (0.989 , 0.993) | <0.001 |
| Day 14 CALLY index | 0.993 (0.991 , 0.995) | <0.001 |
| **Male** | 1.496 (1.232 , 1.818) | <0.001 |
| **Age, years** | 1.016 (1.009 , 1.023) | <0.001 |
| BMI, kg/m² | 0.954 (0.922 , 0.988) | 0.008 |
| Gram-positive bacteria | 1.013 (0.835 , 1.230) | 0.893 |
| Gram-negative bacteria | 0.933 (0.785 , 1.110) | 0.433 |
| Fungus | 1.101 (0.914 , 1.326) | 0.312 |
| Virus | 1.508 (1.160 , 1.960) | 0.002 |
| Multiple infections | 0.810 (0.678 , 0.967) | 0.20 |
| Lower respiratory tract infection | 1.976 (1.621 , 2.408) | <0.001 |
| Gastrointestinal infection | 0.293 (0.157 , 0.548) | <0.001 |
| Intra-abdominal infection | 1.406 (1.157 , 1.709) | <0.001 |
| Urinary tract infection | 0.336 (0.259 , 0.436) | <0.001 |
| Bacteremia | 0.703 (0.579 , 0.854) | <0.001 |
| Skin and soft tissue infection | 0.945 (0.667 , 1.338) | 0.749 |
| CRRT | 2.011 (1.672 , 2.419) | <0.001 |
| Vasoactive agents | 2.595 (2.055 , 3.277) | <0.001 |
| Fluid resuscitation | 0.494 (0.415 , 0.588) | <0.001 |
| Corticosteroid therapy | 1.742 (1.462 , 2.076) | <0.001 |
| Mechanical ventilation | 8.617 (6.597 , 11.255) | <0.001 |
| Diabetes mellitus | 0.958 (0.804 , 1.140) | 0.627 |
| Hypertension | 0.896 (0.741 , 1.082) | 0.253 |
| Heart failure | 1.481 (1.233 , 1.777) | <0.001 |
| Atrial fibrillation | 1.161 (0.937 , 1.439) | 0.172 |
| Renal disease | 1.015 (0.842 , 1.224) | 0.877 |
| Chronic Liver disease | 1.426 (1.176 , 1.730) | <0.001 |
| COPD | 1.335 (0.959 , 1.858) | 0.087 |
| CAD | 1.035 (0.819 , 1.310) | 0.772 |
| Stroke | 0.944 (0.760 , 1.172) | 0.602 |
| Malignancy | 1.517 (1.253 , 1.837) | <0.001 |
| Respiratory failure | 5.845 (4.588 , 7.447) | <0.001 |
| ARDS | 1.818 (1.444 , 2.288) | <0.001 |
| Pneumonia | 2.393 (1.942 , 2.949) | <0.001 |
| Arterial partial pressure of carbon dioxide (PaCO₂), mmHg | 1.000 (0.999 , 1.001) | 0.831 |
| Base excess (BE), mmol/L | 0.961 (0.946 , 0.977) | <0.001 |
| Bicarbonate (HCO₃⁻), mmol/L | 0.974 (0.956 , 0.992) | 0.004 |
| Lactate (Lac), mmol/L | 1.062 (1.042 , 1.082) | <0.001 |
| C-reactive protein (CRP), mg/L | 0.998 (0.997 , 0.999) | 0.002 |
| White blood cell (WBC) count, ×10⁹/L | 0.983 (0.972 , 0.993) | 0.001 |
| Lymphocyte count, ×10⁹/L | 0.985 (0.871 , 1.113) | 0.808 |
| Hemoglobin (Hb), g/L | 0.994 (0.990 , 0.997) | <0.001 |
| Platelet (Ptl) count, ×10⁹/L | 1.001 (1.001 , 1.002) | 0.003 |
| Prothrombin time (PT), s | 1.033 (1.024 , 1.041) | <0.001 |
| International normalized ratio (INR) | 1.020 (0.976 , 1.065) | 0.389 |
| Activated partial thromboplastin time (APTT), s | 1.008 (1.003 , 1.013) | <0.001 |
| D-dimer, mg/L FEU | 1.008 (1.003 , 1.013) | 0.195 |
| Triglycerides (TG), mmol/L | 0.883 (0.788 , 0.989) | 0.032 |
| Total cholesterol, mmol/L | 1.014 (0.987 , 1.042) | 0.298 |
| Alanine aminotransferase (ALT), U/L | 1.000 (1.000, 1.000) | 0.098 |
| Albumin (Alb), g/L | 0.970 (0.955 , 0.984) | <0.001 |
| Total bilirubin (TBil), μmol/L | 1.002 (1.001 , 1.003) | 0.020 |
| Blood urea nitrogen (BUN), mmol/L | 1.020 (1.013 , 1.027) | <0.001 |
| Creatinine, μmol/L | 1.000 (1.000 , 1.001) | 0.378 |
| Calcium (Ca²⁺), mmol/L | 1.019 (1.006, 1.032) | 0.005 |
| Potassium (K⁺), mmol/L | 1.001 (0.986 , 1.017) | 0.863 |
| Procalcitonin (PCT), ng/mL | 0.991 (0.988 , 0.994) | <0.001 |
| Phosphorus (P), mmol/L | 0.998 (0.993 , 1.004) | 0.560 |
| Magnesium (Mg²⁺), mmol/L | 1.019 (1.005 , 1.033) | 0.009 |
| SAPS II | 1.034 (1.029 , 1.039) | <0.001 |
| APACHE II score | 1.082 (1.071 , 1.094) | <0.001 |
| SOFA score | 1.203 (1.176 , 1.231) | <0.001 |
| Glasgow coma scale (GCS) score | 0.858 (0.843 , 0.874) | <0.001 |

**Notes:** CI, confidence interval; HR, hazard ratio.

**Table A5. Multivariable Cox regression analyses of the association between Day 3 CALLY index and mortality in patients with SA-AKI.**

(**A**) 60-day mortality,

| **Variable** | **Model 1** | | **Model 2** | | **Model 3** | |
| --- | --- | --- | --- | --- | --- | --- |
|  | **HR (95% CI)** | ***P* value** | **HR (95% CI)** | ***P* value** | **HR (95% CI)** | ***P* value** |
| **Continuous variable** | 0.996  (0.994–0.998) | <0.001 | 0.997  (0.994–0.999) | 0.001 | 0.997  (0.995–0.999) | 0.002 |
| **Quartile** |  | | | | | |
| **Q1** | 1 |  | 1 |  | 1 |  |
| **Q2** | 0.637  (0.507–0.801) | <0.001 | 0.644  (0.512–0.809) | <0.001 | 0.808  (0.639–1.022) | 0.076 |
| **Q3** | 0.623  (0.496–0.783) | <0.001 | 0.631  (0.502–0.793) | <0.001 | 0.658  (0.519–0.833) | <0.001 |
| **Q4** | 0.456  (0.357–0.583) | <0.001 | 0.498  (0.389–0.638) | <0.001 | 0.589  (0.453–0.764) | <0.001 |
| ***P* for trend** |  | 0.007 |  | 0.011 |  | 0.021 |

(**B**) 365-day mortality,

| **Variable** | **Model 1** | | **Model 2** | | **Model 3** | |
| --- | --- | --- | --- | --- | --- | --- |
|  | **HR (95% CI)** | ***P* value** | **HR (95% CI)** | ***P* value** | **HR (95% CI)** | ***P* value** |
| **Continuous variable** | 0.996  (0.994–0.998) | <0.001 | 0.997  (0.995–0.999) | 0.002 | 0.997  (0.995–0.999) | 0.002 |
| **Quartile** |  | | | | | |
| **Q1** | 1 |  | 1 |  | 1 |  |
| **Q2** | 0.632  (0.508–0.787) | <0.001 | 0.641  (0.514–0.798) | <0.001 | 0.807  (0.644–1.011) | 0.062 |
| **Q3** | 0.599  (0.480–0.748) | <0.001 | 0.608  (0.487–0.760) | <0.001 | 0.644  (0.512–0.810) | <0.001 |
| **Q4** | 0.453  (0.358–0.573) | <0.001 | 0.494  (0.390–0.627) | <0.001 | 0.587  (0.456–0.754) | <0.001 |
| ***P* for trend** |  | 0.010 |  | 0.015 |  | 0.027 |

(**C**)In-hospital mortality.

| **Variable** | **Model 1** | | **Model 2** | | **Model 3** | |
| --- | --- | --- | --- | --- | --- | --- |
|  | **HR (95% CI)** | ***P* value** | **HR (95% CI)** | ***P* value** | **HR (95% CI)** | ***P* value** |
| **Continuous variable** | 0.996  (0.994–0.998) | <0.001 | 0.997  (0.994–0.999) | 0.001 | 0.997  (0.995–0.999) | 0.001 |
| **Quartile** |  | | | | | |
| **Q1** | 1 |  | 1 |  | 1 |  |
| **Q2** | 0.630  (0.505–0.787) | <0.001 | 0.641  (0.513–0.800) | <0.001 | 0.806  (0.641–1.013) | 0.003 |
| **Q3** | 0.597  (0.477–0.747) | <0.001 | 0.607  (0.484–0.760) | <0.001 | 0.641  (0.508–0.808) | <0.001 |
| **Q4** | 0.437  (0.343–0.556) | <0.001 | 0.479  (0.375–0.610) | <0.001 | 0.569  (0.440–0.736) | <0.001 |
| ***P* for trend** |  | 0.010 |  | 0.014 |  | 0.027 |

**Notes:**

Model 1: unadjusted.

Model 2: adjusted for: sex, age, hypertension, diabetes mellitus.

Model 3: fully adjusted (Model 2 + chronic liver disease, CRRT, fluid resuscitation, APACHE II score, procalcitonin, lactate, gastrointestinal infection, fungal infection).

**Table A6. Results of the moderated mediation analysis for the association between age, Day 3 CALLY index and mortality in SA-AKI**

(**A**) 30-day mortality,

|  | **Coefficient (95% CI)** | ***P* value** |
| --- | --- | --- |
| **Total effect** | 0.0504 (0.0142–0.0875) | 0.004 |
| **Mediation effect** | 0.0120 (0.0036–0.0234) | <0.001 |
| **Direct effect** | 0.0384 (0.0035–0.0763) | 0.030 |
| **Proportion mediated** | 0.2376 (0.0741–0.7644) | 0.004 |
| **Interaction *P* value** | 0.6040 | |

(**B**) 60-day mortality,

|  | **Coefficient (95% CI)** | ***P* value** |
| --- | --- | --- |
| **Total effect** | 0.0581 (0.0234–0.0917) | <0.001 |
| **Mediation effect** | 0.0154 (0.0054–0.0278) | <0.001 |
| **Direct effect** | 0.0427 (0.0114–0.0788) | 0.018 |
| **Proportion mediated** | 0.2654 (0.0953–0.6313) | <0.001 |
| **Interaction *P* value** | 0.7340 | |

(**C**) 365-(**B**) 60-day mortality,day mortality,

|  | **Coefficient (95% CI)** | ***P* value** |
| --- | --- | --- |
| **Total effect** | 0.0576 (0.0230–0.0898) | 0.002 |
| **Mediation effect** | 0.0149 (0.0053–0.0269) | <0.001 |
| **Direct effect** | 0.0418 (0.0109–0.0764) | 0.010 |
| **Proportion mediated** | 0.2633 (0.0891–0.5927) | 0.002 |
| **Interaction *P* value** | 0.6960 | |

(**D**)In-hospital mortality,

|  | **Coefficient (95% CI)** | ***P* value** |
| --- | --- | --- |
| **Total effect** | 0.0547 (0.0203–0.0875) | <0.001 |
| **Mediation effect** | 0.0157 (0.0056–0.0280) | <0.001 |
| **Direct effect** | 0.0390 (0.0075–0.0738) | 0.020 |
| **Proportion mediated** | 0.2869 (0.0100–0.7162) | <0.001 |
| **Interaction *P* value** | 0.5220 | |

**Note:** Mediation analysis was performed using the R package mediation with 1,000 bootstrap samples, adjusted for sex, hypertension, diabetes mellitus, chronic liver disease, CRRT, fluid resuscitation, APACHE II score, procalcitonin, lactate, gastrointestinal infection, and fungal infection. The Interaction *P* value tests for the significance of the age × CALLY index interaction term in the moderated mediation model.

**Table A7. Complete-case sensitivity analysis for the association between time-varying CALLY index trajectories and 30-day mortality in SA-AKI**

| **Variable** | **HR (95% CI)** | ***P* value** |
| --- | --- | --- |
| Age,years | 1.010 (1.003–1.017) | 0.0046 |
| Sex | 1.328 (1.098–1.607) | 0.0035 |
| Hypertension | 0.913 (0.759–1.098) | 0.3269 |
| Diabetes mellitus | 0.819 (0.674–0.996) | 0.0450 |
| Chronic liver disease | 1.058 (0.866–1.293) | 0.5821 |
| CRRT | 1.343 (1.114–1.619) | 0.0021 |
| Fluid resuscitation | 0.649 (0.541–0.778) | <0.001 |
| APACHE II score | 1.077 (1.064–1.090) | <0.001 |
| Procalcitonin | 0.993 (0.990–0.996) | <0.001 |
| Lactate | 1.040 (1.020–1.060) | 0.0010 |
| Gastrointestinal infection | 0.452 (0.264–0.774) | 0.0038 |
| Fungal infection | 0.843 (0.700–1.015) | 0.0720 |
| Time-varying variables |  | |
| CALLY index | 0.996 (0.995–0.997) | <0.001 |

**Notes:** CI, confidence interval; CRRT, continuous renal replacement therapy; APACHE II, Acute Physiology and Chronic Health Evaluation II; HR, hazard ratio; SA-AKI, sepsis-associated acute kidney injury.

**Table A8.** Model comparison using Deviance Information Criterion (DIC)

| **Model** | **Description** | **DIC** |
| --- | --- | --- |
| **Model 0** | Baseline CALLY + all covariates (without longitudinal dynamics) | 6508.63 |
| **Model 1** | Baseline CALLY + all covariates + longitudinal CALLY dynamic features (slope, mean, range) | 6052.66 |
| **ΔDIC** | Model 1 − Model 0 | −455.97 |

**Notes:** DIC, Deviance Information Criterion; CALLY, C-reactive protein/albumin-lymphocyte index. All models were adjusted for the same set of covariates: age, sex, lactate, PCT, APACHE II score, CRRT, fluid resuscitation, hypertension, diabetes mellitus, chronic liver disease, fungal infection, and gastrointestinal infection.

Lower DIC values indicate better model fit; ΔDIC < −5 was considered strong evidence of improvement.

****Figure A1. Kaplan-Meier survival curves for mortality in SA-AKI stratified by Day 1 CALLY index quartiles.****
(A) 30-day, (B) 60-day, (C) 365-day. **Quartiles** **cutoffs were defined as follows:** Q1 ≤5.92, Q2 5.92–12.25, Q3 12.25–28.69, **and** Q4 > 28.69. Survival distributions did not differ significantly across quartiles (log-rank *P* > 0.05).

****Figure A2. Kaplan-Meier survival curves for mortality in SA-AKI stratified by Day 7 CALLY index quartiles.****
(A) 30-day, (B) 60-day, (C) 365-day. **Quartiles** **cutoffs were defined as follows:** Q1 ≤17.84, Q2 17.84–42.74, Q3 42.74–139.95, **and** Q4 >139.95. Survival distributions did not differ significantly across quartiles (log-rank *P*<0.001)

****Figure A3. Kaplan-Meier survival curves for mortality in SA-AKI stratified by Day 14 CALLY index quartiles.****
(A) 30-day, (B) 60-day, (C) 365-day. **Quartiles** **cutoffs were defined as follows:** Q1 ≤18.36, Q2 18.36–56.80, Q3 56.80–302.90, **and** Q4 >302.90. Survival distributions did not differ significantly across quartiles (log-rank *P*<0.001)
